# In this month’s Bulletin

**DOI:** 10.2471/BLT.18.001118

**Published:** 2018-11-01

In the editorial section, Michelle McIsaac et al. (730) discuss the findings of a randomized trial on results-based financing, published in this issue of the *Bulletin*. Corrado Barbui et al. (731) describe the lessons learned from a national reform of mental health care provision in Italy. 

Sophie Cousins (734–735) reports from Bangladesh on efforts to improve the distribution and capacity of the health workforce. Christian Ntizimira talks to Tatum Anderson (736–737) about the challenges of adapting models of palliative care to an African setting. 

## Burkina Faso

### Aiming for national estimates

Nicolas Meda et al. (750–759) measure the seroprevalence of Hepatitis B and C virus.

## Ghana, Kenya, Mauritius, Morocco, Namibia, Senegal, Tunisia and United Republic of Tanzania

### Establishing the prevalence of childhood obesity

Adama Diouf et al. (772–781) compare body mass index with deuterium dilution. 

## Indonesia, Peru, Romania, South Africa

### Co-morbidities: tuberculosis and diabetes

Daniel Grint et al. (738–749) assess the accuracy of diabetes screening methods in people with tuberculosis.

## Zambia

### Linking pay and performance

Wu Zeng et al. (760–771) test different ways of implementing results-based financing.

## Global

### Ensuring a well-stocked pharmacy

Anne Paschke et al. (782–791) describe measures to increase transparency and accountability in national pharmaceutical systems.

### Beyond infectious disease threats

Amrita Saha & George Alleyne (792–793) argue that noncommunicable diseases should be considered as a global health security issue.

### Are people exercising enough?

Justin J Lang et al. (794–796) argue for measures of cardiorespiratory fitness.

